# Prediction of Droplet Production Speed by Measuring the Droplet Spacing Fluctuations in a Flow-Focusing Microdroplet Generator

**DOI:** 10.3390/mi10120812

**Published:** 2019-11-25

**Authors:** Wen Zeng, Dong Xiang, Hai Fu

**Affiliations:** 1 Department of Fluid Control and Automation, Harbin Institute of Technology, Harbin 150001, China; fuhai19890909@163.com; 2 State Key Laboratory of Fluid Power and Mechatronic Systems, Zhejiang University, Hangzhou 310027, China

**Keywords:** droplet, production speed, spacing, fluctuations, flow-focusing

## Abstract

In a flow-focusing microdroplet generator, by changing the flow rates of the two immiscible fluids, production speed can be increased from tens to thousands of droplets per second. However, because of the nonlinearity of the flow-focusing microdroplet generator, the production speed of droplets is difficult to quantitatively study for the typical flow-focusing geometry. In this paper, we demonstrate an efficient method that can precisely predict the droplet production speed for a wide range of fluid flow rates. While monodisperse droplets are formed in the flow-focusing microchannel, droplet spacing as a function of time was measured experimentally. We discovered that droplet spacing changes periodically with time during each process of droplet generation. By comparing the frequency of droplet spacing fluctuations with the droplet production speed, precise predictions of droplet production speed can be obtained for different flow conditions in the flow-focusing microdroplet generator.

## 1. Introduction

The design of microdroplet generators requires that a wide range of droplet production speeds can be tuned for different experiments. In particular, controlling and predicting the production speed of droplets is of significant importance for droplet-based microfluidic systems [1,2,3,4] and applications in experiments, such as chemical reactions [5], nanoparticle synthesis [6], and biomedical diagnosis [7,8,9]. As such, an experimental method that can achieve high accuracy for droplet production speed prediction is quite meaningful for improving the applications of droplet microfluidic systems [10,11,12]. 

According to the literature, in a flow-focusing microdroplet generator, the principle of droplet formation has been studied both theoretically and experimentally [13,14,15]. For various flow conditions, it has been observed that both the size and the production speed of droplets vary nonlinearly with the flow-rate ratio of two immiscible fluids [16,17,18]. Especially for high capillary numbers, the production speed of droplets also varies with the viscosity of fluids for a specific flow-rate ratio [19,20]. Due to the inherent nonlinearity between droplet production speed and flow-rate ratio in a flow-focusing microdroplet generator, the production speed of droplets is quite difficult to accurately predict [21]. More importantly, droplet production speed is mostly obtained by online detection, while monodisperse droplets are generated in a flow-focusing microdroplet generator [22,23]. While some new methods of droplet formation have been provided for monodisperse droplet production [24,25,26], to the best of our knowledge, the nonlinear relationship between droplet production speed and fluid flow rate is mainly qualitatively discussed in experiments of droplet generation in the flow-focusing microchannel [27]. 

In this paper, we provide an efficient method to precisely predict droplet production speed for a wide range of fluid flow rates in a flow-focusing microdroplet generator. Different viscosities of silicone oil were chosen for experimental measurements of droplet production speed. The nonlinear relationship between droplet production speed and flow-rate ratio was tested experimentally, and the effects of fluid viscosity on this nonlinear relationship of droplet formation are discussed. While monodisperse droplets are generated in a flow-focusing microchannel, it can be observed that droplet spacing changes periodically with time during each process of droplet generation. Most importantly, by increasing the droplet production speed from approximately 10 to 100 per second, the comparison between the frequency of droplet spacing fluctuations and the production speed of droplets was obtained experimentally. As a result, from experimental measurements of the periodic fluctuations of droplet spacing, the prediction precision of the production speeds of droplets can be quantitatively studied for various flow conditions in the flow-focusing microdroplet generator.

## 2. Experimental Setup

**Figure 1:**
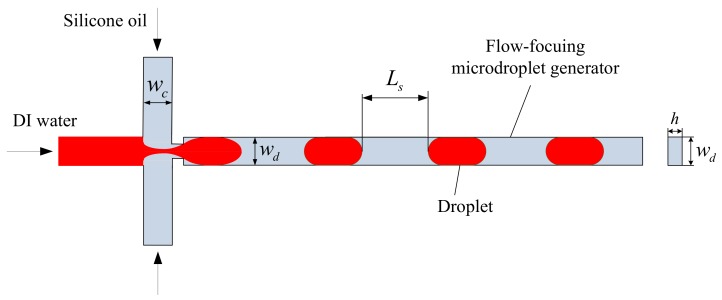
Principle of droplet formation in a Flow-focusing microdroplet generator.

A flow-focusing microdroplet generator was designed for measuring the periodic fluctuations of droplet spacing as monodisperse droplets were produced. Meanwhile, the frequency of droplet spacing fluctuations was quantified experimentally, which could be used to precisely predict the production speed of droplets. Figure 1 shows the principle of droplet formation in a flow-focusing microdroplet generator. 

Here, $w_{c}$ is the channel width of the continuous phase, $w_{d}$ is the channel width of the disperse phase, and h is the channel height; the geometrical parameters of the microchannel are specified as $w_{c}=w_{d}=100$ μm and h=50 μm. For droplet generation, we chose deionized (DI) water as the disperse phase and silicone oil as the continuous phase. The viscosity of the DI water was $\mu_{d}=1$ cP and the viscosity of the silicone oil span from 10 to 200 cP. The Sylgard 184 (Dow Corning, Michigan, USA) was used for fabricating the polydimethylsiloxane (PDMS) microchannels. The mixing ratio of the base polymer to the curing agent was 10 to 1, and the material was cured in a heater at 80 °C for 8 h. As soon as the PDMS microchannel is prepared, the oxygen plasma should be applied to the PDMS microchannel for approximately 30 s. Since the oxygen plasma was applied before bonding, the PDMS microchannel could be bonded to a glass slide substrate and kept in the heater for about 4 h. Finally, a flow-focusing microdroplet generator could be fabricated. In this paper, for the specific geometry of a flow-focusing microdroplet generator, all the experiments of monodisperse droplet production were performed at low capillary numbers ($C_{a}\leq 0.1$).

The commercial syringe pump (Harvard Apparatus Standard Infuse/Withdraw PHD Ultra Syringe Pump, Harvard Apparatus, Holliston, MA, USA) was used to supply the flow rates of the continuous and disperse phases. While monodisperse droplets were formed in the flow-focusing microdroplet generator, the production rates of droplets could be verified by changing the flow rates of the two phases. In addition, a high-speed camera (Phantom v9.1, Vision Research, Wayne, NJ, USA) was chosen to take images of the droplet generation, and the speed of image acquisition of droplet formation was specified as 1000 fps. Using the method of image processing, the image of the droplet generation was analyzed by the image processing toolbox of the Matlab software (R2019b, MathWorks, Natick, MA, USA). By calculating the pixels of the droplet spacing along the microchannel, the time-varying droplet spacing could be measured experimentally, while monodisperse droplets were produced within the microchannel. For our experiments of droplet formation, the production rates of droplets spanned from approximately 10 to 100 per second; therefore, the sampling frequency of the droplet spacing acquisition was much higher than the production speed of the droplets. Consequently, the periodic fluctuations of droplet spacing could be easily captured, while monodisperse droplets were produced in the flow-focusing microdroplet generator. 

## 3. Results and Discussion

### 3.1. Experimental Measurements of Droplet Production Speed

**Figure 2:**
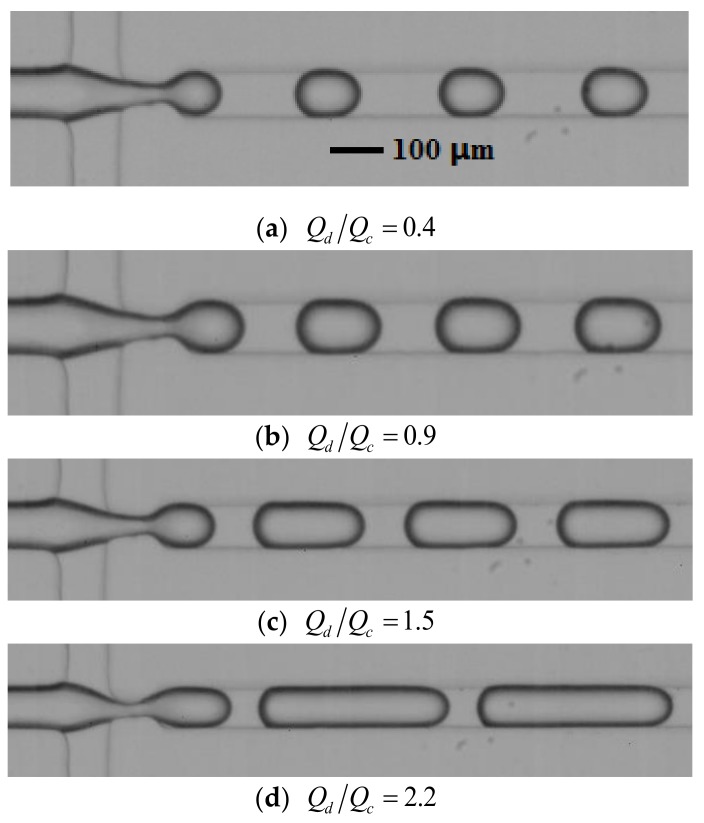
Monodisperse droplet generation in the flow-focusing microdroplet generator.

For the flow-focusing microdroplet generator, three different viscosities of the silicone oil ($\mu_{c}=10,\;100,\;200$ cP) were chosen for the experimental measurements of droplet production speed, $f_{d}$. While the flow-rate ratio, $Q_{d}/Q_{c}$, of the two immiscible fluids increased from 0.2 to 2.4, monodisperse droplets could be generated in the flow-focusing microdroplet generator, as shown in Figure 2.

**Figure 3:**
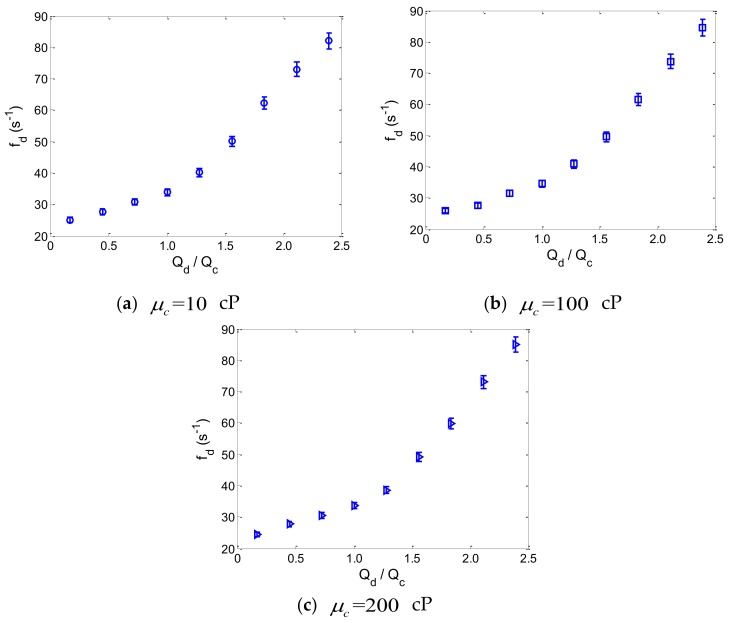
Experimental measurements of droplet production speed as a function of the flow-rate ratio in the flow-focusing microdroplet generator. The error bars represent the standard deviation of seven separate measurements of the droplet production speed for a specific flow-rate ratio.

Particularly, while monodisperse droplets were produced in the microchannel, the relationship between droplet production speed, $f_{d}$, and the flow-rate ratio, $Q_{d}/Q_{c}$, was measured experimentally, as shown in Figure 3. The error bars represent the standard deviation of seven separate measurements of droplet production speed for a specific flow-rate ratio of the two phases. 

From the experimental results, it can be observed that the droplet production speed varies nonlinearly with the flow-rate ratio for different viscosities of the fluids. More importantly, since all of the experiments of monodisperse droplet production were conducted at low capillary numbers ($C_{a}\leq 0.1$), this nonlinear relationship between droplet production speed and flow-rate ratio was mainly determined by the geometrical parameters of the flow-focusing microdroplet generator, and the influence of fluid viscosity on this nonlinear relationship can be neglected [19]. Consequently, because of the nonlinearity of flow-focusing, the production speed of droplets is quite difficult to accurately predict for different flow conditions within the flow-focusing microchannel.

### 3.2. Precise Prediction of Droplet Production Speed

**Figure 4:**
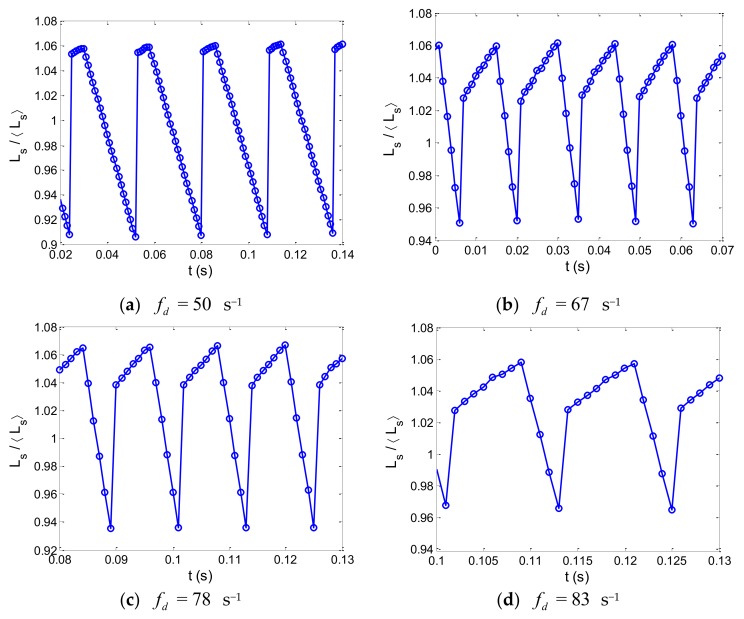
Dimensionless droplet spacing as a function of time for a fixed droplet production speed in the flow-focusing microdroplet generator. The viscosity of the silicone oil is specified as $\mu_{c}\;=\;10$ cP.

For the flow-focusing microdroplet generator, a wide range of droplet production speeds can be obtained by changing the flow-rate ratio of the two phases. Droplet spacing as a function of time was tested experimentally for the different production speeds of the droplets. Here, $L_{s}$ is defined as time-varying droplet spacing, and $\langle L_{s}\rangle$ is defined as the average droplet spacing for each period of the droplet formation process. We used the nondimensional droplet spacing, $L_{s}/\langle L_{s}\rangle$, to simplify the analysis of the periodic fluctuations induced by the droplet generation progress. Figure 4 shows the dimensionless droplet spacing, $L_{s}/\langle L_{s}\rangle$, as a function of time for a fixed droplet production speed, while monodispersed droplets were generated in the microchannel. Here, the viscosity of the silicone oil is specified as $\mu_{c}\;=\;10$ cP.

**Figure 5:**
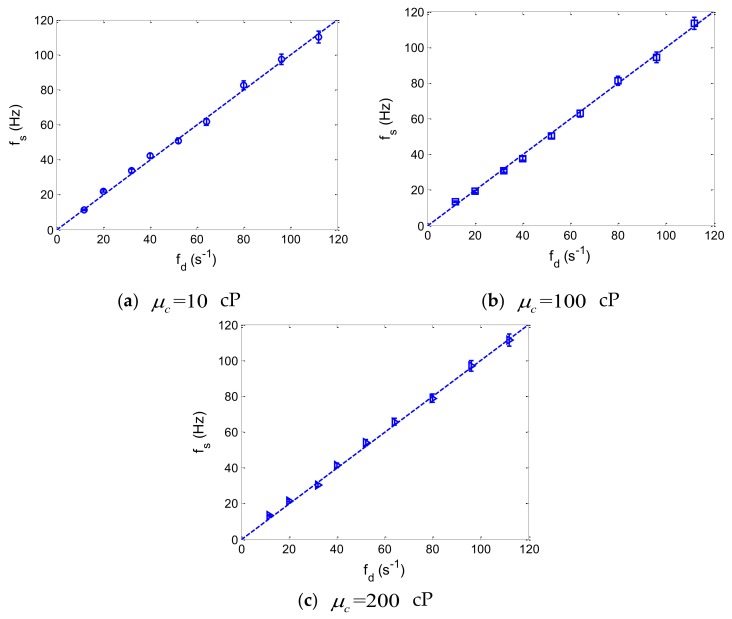
Comparison between the frequency of droplet spacing fluctuations and droplet production speed while monodisperse droplets are produced in the flow-focusing microdroplet generator. The error bars represent the standard deviation of seven separate measurements of the periodic fluctuation frequency for a specific droplet production speed.

Based on the experimental measurements, we note that droplet spacing,$L_{s}$, changes periodically with time during the dynamic process of droplet generation. Here, we define $f_{s}$ as the frequency of periodic fluctuations of droplet spacing, $L_{s}$, while monodisperse droplets are generated in the flow-focusing microchannel. Particularly, the frequency, $f_{s}$, of the droplet spacing fluctuations is associated with droplet production speed, $f_{d}$. For the typical geometry of the flow-focusing, three different viscosities of the silicone oil ($\mu_{c}\;=\;10,\;100,\;200$ cP) were chosen for the experimental measurements of the periodic fluctuations of droplet spacing, as monodisperse droplets were formed in the microchannel. Additionally, by using fast Fourier transform on the experimental data of the time-varying droplet spacing, the frequency of droplet spacing fluctuations could be accurately calculated. Figure 5 shows the comparison between the frequency of droplet spacing fluctuations and droplet production speed, while monodisperse droplets are produced in the flow-focusing microdroplet generator. The error bars represent the standard deviation of seven separate measurements of the periodic fluctuation frequency for a specific droplet production speed. 

From Figure 5, it can be observed that while droplet production speed increases from approximately 10 to 100 droplets per second, the frequency of droplet spacing fluctuations is consistent with the production speed of droplets. For different viscosities of the fluids, good agreement is shown between the frequency of the droplet spacing fluctuations and the droplet production speed. As a result, as long as monodisperse droplets can be generated in the flow-focusing microdroplet generator, the production speeds of droplets can be precisely predicted by measuring the periodic fluctuations of the droplet spacing for various flow conditions. 

## 4. Conclusions 

In a flow-focusing microdroplet generator, we demonstrate an efficient method that can precisely predict the droplet production speed for a wide range of fluid flow rates. While monodisperse droplets were being formed in the flow-focusing microchannel, we discovered that the droplet production speed varies nonlinearly with the flow-rate ratio, and especially for a fixed flow-rate ratio, the droplet spacing changes periodically with time during each process of droplet generation. Most importantly, as the droplet production speed varies from approximately 10 to 100 per second, the frequency of the droplet spacing fluctuations coincides with the production speed of droplets. As a result, from experimental measurements of the periodic fluctuations of the droplet spacing, it is validated that the production speed of droplets can be precisely predicted for various flow conditions within the flow-focusing microdroplet generator.
